# 1763. Attendance and Learning Outcomes of Statewide Infection Prevention and Control Education and Support Webinars Initiative During the COVID-19 Pandemic: The Nebraska Experience

**DOI:** 10.1093/ofid/ofad500.1594

**Published:** 2023-11-27

**Authors:** Rebecca Martinez, Mounica Soma, Josette McConville, Laura "Kate" Tyner, Matthew Donahue, Muhammad Salman Ashraf, Daniel M Brailita

**Affiliations:** Nebraska Infection Control Assessment and Promotion Program, Nebraska Medicine, Omaha Nebraska, Omaha, Nebraska; Nebraska Medicine, Omaha, Nebraska; Nebraska Infection Control Assessment and Promotion Program, Nebraska Medicine, Omaha Nebraska, Omaha, Nebraska; Nebraska Medicine, Omaha Nebraska, Omaha, Nebraska; Division of Public Health, Department of Health and Human Services, Lincoln, Nebraska, Lincoln, Nebraska; Division of Public Health, Department of Health and Human Services, Lincoln, Nebraska;Division of Infectious Diseases, University of Nebraska Medical Center, Omaha Nebraska;Nebraska Infection Control Assessment and Promotion Program, Nebraska Medicine, Omaha Nebraska, Omaha, Nebraska; Division of Infectious Diseases, University of Nebraska Medical Center, Omaha Nebraska;Nebraska Infection Control Assessment and Promotion Program, Nebraska Medicine, Omaha Nebraska, Omaha, Nebraska

## Abstract

**Background:**

In October 2020, the Nebraska Infection Control Assessment and Promotion program (ICAP), funded by the Nebraska Department of Health and Human Services (DHHS) through a CDC grant, launched a statewide initiative providing infection prevention and control (IPC) education and guidance through weekly webinars to long-term care (LTC) facilities. In March 2021, this was expanded to include a twice monthly webinar for acute care (AC) and outpatient facilities. ICAP infection preventionists (IPs) and medical directors developed the content of webinars in collaboration with Nebraska DHHS. Webinar recordings and slides were posted for reference. Webinar reminders were shared with facilities through the ICAP email distribution list. We investigated factors driving attendance and learning outcomes for this initiative.

**Methods:**

Attendance was recorded for each webinar and compared between LTC and AC considering webinar focus and known denominators of Nebraska LTC and acute facilities. A comprehensive survey regarding learning outcomes and factors driving participation was sent to webinar contacts in early 2023. Responses from IPs working in AC and LTC were compared by independent two-sample T-Test with Pooled and Satterthwaite distribution using SAS version 9.04.01

**Results:**

On average, 69 (range 42 to 110) facility IP leaders attended AC webinars and 160 (range 123 to 526) attended LTC webinars since the inception. Of the 96 AC and 429 LTC operational facilities, 50% AC and 25% LTC facilities responded to the survey. Of the 51 AC and 170 LTC respondents, IPs constituted 86% (n=44) and 25% (n=42) respectively. LTC IPs were more likely than AC IPs to join the webinars to learn about changes in regulatory requirements and national IPC guidance. More than 60% of IPs in both settings implemented a new policy or procedure based on the webinar, with LTC IP’s implementing 5% more general IP and 9% more COVID-19 specific policies. 83.3 % of LTC IPs and 52.3% of the AC IPs reported attending almost all webinars. IPs self-rated learning outcomes were high (AC 4.5-4.6 and LTC 4.3-4.6 on a 1-5 scale)

**Figure:**
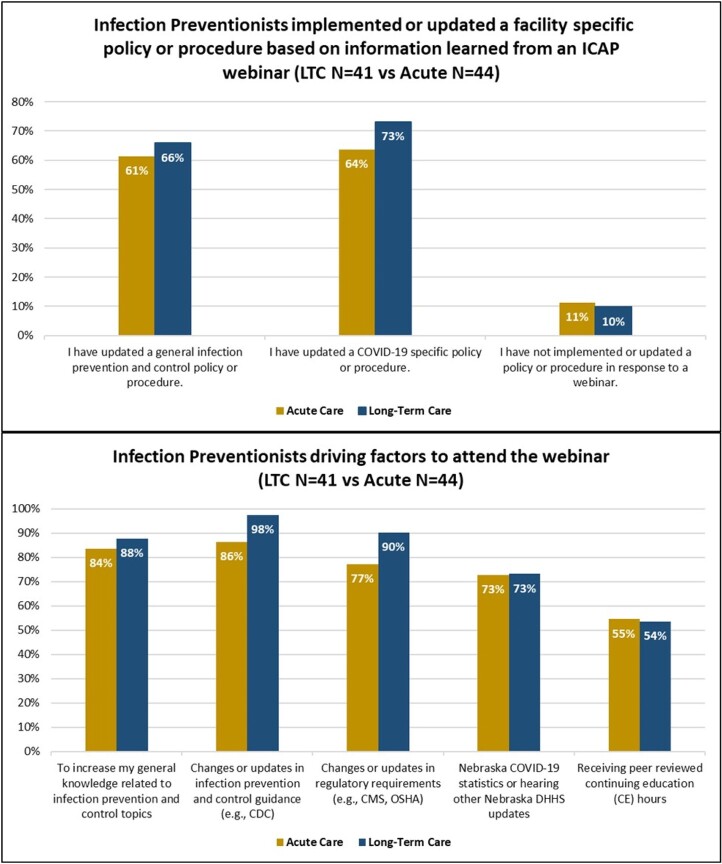

**Figure:**
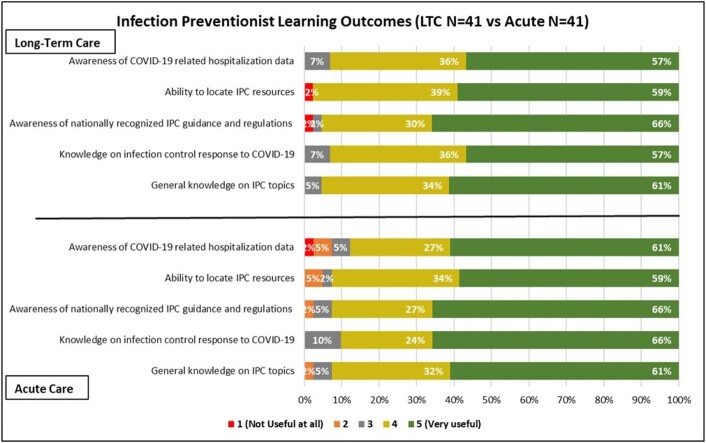

**Conclusion:**

A statewide initiative to provide infection prevention education and guidance was well received by Nebraska facilities and led to just-in-time policy and procedure changes during COVID-19 pandemic.

**Figure:**
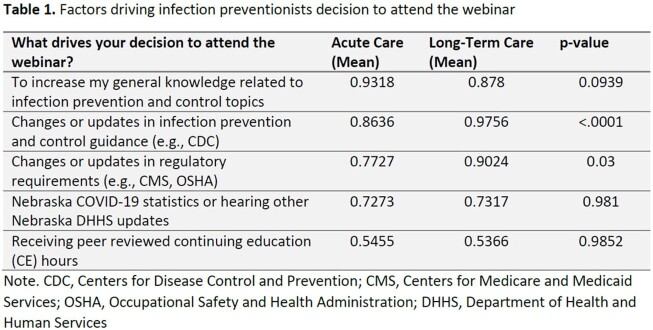

**Disclosures:**

**Muhammad Salman Ashraf, MBBS**, Merck: Grant/Research Support

